# rs965513 polymorphism as a common risk marker is associated with papillary thyroid cancer

**DOI:** 10.18632/oncotarget.9324

**Published:** 2016-05-12

**Authors:** Fang Wang, Dehui Yan, Xu Ji, Jun Han, Meijun Chen, Hong Qiao, Shaojun Zhang

**Affiliations:** ^1^ College of Bioinformatics Science and Technology, Harbin Medical University, Harbin, 150081, China; ^2^ Department of Otolaryngology, The First Affiliated Hospital of China Medical University, Shenyang, 110001, China; ^3^ Department of Endemic Disease, the Second Affiliated Hospital, Harbin Medical University, Harbin, 150086, China

**Keywords:** thyroid cancer, genome-wide association studies, FOXE1, rs965513, meta-analysis

## Abstract

Papillary thyroid cancer (PTC) is the most common type of thyroid cancer. With the rapid development of genome-wide association studies (GWAS), many genome variants associated with susceptibility to PTC have been identified, including the single nucleotide polymorphism rs965513 (9q22.33) near FOXE1. To evaluate the association between rs965513 and PTC in different ethnicities and countries, we conducted a meta-analysis using relatively large-scale samples from 23 studies (*N* = 163,136; 20,736 cases and 142,400 controls) by searching the PubMed and Google Scholar databases. Significant heterogeneity caused by different populations among the selected studies was observed. The A allele of rs965513 polymorphism was shown to be highly associated with risk of thyroid cancer, with odds ratios of 1.58 (95% CI 1.32–1.90) in all populations, 1.65 (95% CI 1.31–2.07)) in Caucasian populations and 1.49 in Asian populations. Compared to the dominant and recessive models, we observed the highest odds ratio (OR = 2.80, 95% CI 2.12–3.69) in the homozygous model. These results revealed that the rs965513 polymorphism is a risk factor for thyroid cancer

## INTRODUCTION

Thyroid cancer (TC) is the most common malignancy in the endocrine system [1] and the fifth leading malignancy in female patients [2], and papillary thyroid cancer (PTC) is the most common type of thyroid cancer. Recent studies have improved our understanding of the pathogenesis of PTC, including the identification of genetic alterations that activate a common effector pathway involving the RET-Ras-BRAF signaling cascade, as well as other unique chromosomal rearrangements [3]. In addition, a large scale genome-wide association study (GWAS) identified many single-nucleotide polymorphisms (SNPs) that are significantly associated with PTC, such as USF1 on chromosome 1, FOXE1 on chromosome 9, ATM on chromosome 11, NKX2-1 on chromosome 14 [4], XRCC1 on chromosome 19, XRCC3 on chromosome 14 [5], and ALMS1 on chromosome 2 [6].

The rs965513 polymorphism located near FOXE1 was first identified as significantly associated with thyroid cancer by Gudmundsson J in 2009. That study showed that two common variants are associated with thyroid cancer, specifically rs965513 on 9q22.33 (OR = 1.75; *P* = 1.7 × 10^−27^) and rs944289 on 14q13.3 (OR = 1.37; *P* = 2.0 × 10−9) [7]. Currently, rs965513 has shown no association with events involved in the progression of PTC, such as invasion and tumor stage [8]. Due to the heterogeneity of susceptibility to cancer, such that no association between rs965513 and PTC has been observed in the US [9], it is important to investigate whether rs965513 is associated with thyroid cancer risk in all ethnicities and populations. Studies following Gudmundsson J have investigated this association in Cuba, France, Iceland, The US, Spain, China, Poland, Russia, The UK, Germany, Belarus, Portugal and Japan and provide the opportunity to evaluate whether rs965513 can be used as a common marker in other populations [4, 7–18].

Meta-analysis has been described as combining and analyzing quantitative evidence from related studies to produce results based on a whole body of research [19]. Thus, accounting for the importance of the variance and the inconsistency of results, we evaluated the genetic heterogeneity of rs965513 polymorphism in multiple populations by searching the PubMed and Google Scholar database and performed a meta-analysis to achieve a higher statistical power.

## RESULTS

### Literature search

**Figure 1:**
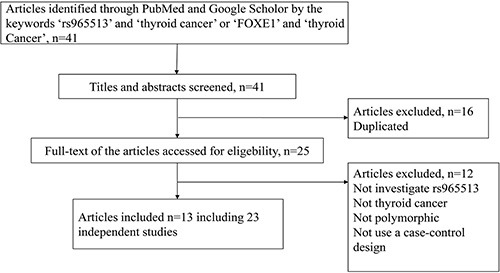
The flow chart for identifying relevant studies

Forty-one articles were selected from the PubMed and Google Scholar databases. Based on the inclusion and exclusion criteria, 13 articles, including 23 independent studies, were included in our analysis. More detailed information about the decision to include or exclude the selected studies can be found in Figure 1. In total, 20,736 cases and 142,400 controls from Cuba, France, Iceland, The US, Spain, China, Poland, Russia, The UK, Germany, Belarus, Portugal and Japan were included in our meta-analysis. Table 1 shows the main characteristics of the included studies: the name of the first author, the year of publication, the population or ethnicity, and the numbers of cases and controls.

**Table 1:** The main characteristics of the included studies

Author	Year	Countryn	Ethnicity	Allele G (freq)		Allele A (freq)	
				Case	Control	Case	Control
Pereda CM	2015	Cuba	Mixed	257 (0.640)	317 (0.750)	149 (0.360)	107 (0.250)
Maillard S	2015	French	Polynesian	235 (0.730)	392 (0.790)	85 (0.270)	104 (0.210)
Gudmundsson J2009	2009	Iceland	Caucasian	590 (0.510)	48206 (0.648)	568 (0.490)	26186 (0.352)
Gudmundsson J2009	2009	US	Caucasian	312 (0.529)	516 (0.671)	276 (0.471)	252 (0.329)
Gudmundsson J2009	2009	Spain	Caucasian	98 (0.556)	1768 (0.658)	80 (0.444)	918 (0.342)
Wang YL	2013	China	Asian	1489 (0.880)	1847 (0.920)	201 (0.120)	163 (0.080)
Liyanarachchi S	2013	US	Caucasian	707 (0.490)	1269 (0.680)	733 (0.510)	613 (0.320)
Liyanarachchi S	2013	Poland	Caucasian	1880 (0.540)	2471 (0.650)	1606 (0.460)	1351 (0.350)
Wei WJ	2015	China	Asian	246 (0.893)	1230 (0.876)	30 (0.107)	170 (0.124)
Takahashi M	2010	Russia	Caucasian	189 (0.487)	238 (0.666)	199 (0.513)	120 (0.334)
Takahashi M	2010	Russia	Caucasian	224 (0.524)	581 (0.648)	204 (0.476)	315 (0.352)
Takahashi M	2010	Russia	Caucasian	279 (0.538)	820 (0.633)	239 (0.462)	476 (0.367)
Jones AM	2012	UK	Caucasian	768 (0.511)	8225 (0.672)	734 (0.489)	4015 (0.328)
Penna-Martinez	2014	Germany	Caucasian	263 (0.549)	348 (0.644)	223 (0.451)	192 (0.356)
Damiola	2014	Byelorussia	Caucasian	78 (0.600)	303 (0.584)	56 (0.400)	153 (0.416)
Tomaz (FNMTC)	2012	Portugal	Caucasian	53 (0.442)	168 (0.646)	67(0.558)	92(0.354)
Tomaz (NMTC)	2012	Portugal	Caucasian	63 (0.394)	168 (0.646)	97 (0.606)	92 (0.354)
Tomaz (all)	2012	Portugal	Caucasian	116 (0.414)	168 (0.646)	164 (0.586)	92 (0.354)
Denny	2011	US	Caucasian	1881 (0.714)	6589 (0.652)	753 (0.286)	3517 (0.348)
Denny	2011	US	Caucasian	1881 (0.714)	7376 (0.655)	753 (0.286)	3888 (0.345)
Matsuse	2011	Japan	Asian	872 (0.910)	5213 (0.943)	86 (0.090)	315 (0.057)
Matsuse	2011	Japan	Asian	692 (0.908)	5213 (0.943)	70 (0.092)	315 (0.057)
Matsuse	2011	Japan	Asian	175 (0.921)	5213 (0.943)	15 (0.079)	315 (0.057)

Mixed in Ethnicity was the mixture of European and African.

### Heterogeneity test

The genetic heterogeneity of the rs965513 polymorphism was evaluated based on the additive, dominant, recessive and homozygous models and the data from the selected studies (Table 2). Significant heterogeneity was observed among these studies. In the additive model (A *vs.* G) and the dominant model (AA + AG *vs.* GG), extreme heterogeneity was observed among the 23 selected studies (additive model: *P* < 0.0001 and I^2^ = 95.4%; dominant model: *P* < 0.0001 and I^2^ = 76.7%). The recessive model (AA *vs.* AG + GG) and the homozygous model (AA *vs.* GG) showed large heterogeneity among the 23 selected studies (recessive model: *P* = 0.0061 and I^2^ = 62.6%; homozygous model: *P* = 0.0016 and I^2^ = 67.8%). There was no significant heterogeneity observed in Asian populations. However, we found significant heterogeneity in Caucasian populations.

**Table 2:** The result of heterogeneity test

Population	Risk model	*Q*	*P*	*I*^2^	95% CI
All	Additive (A *vs* G)	480.41	< 0.0001	95.4%	94.1%–96.4%
	Recessive (AA *vs* AG+GG)	21.41	0.0061	62.6%	23.1%–81.9%
	Dominant (AA +AG *vs* GG)	34.36	< 0.0001	76.7%	55.5%–87.8%
	Homozygous (AA *vs* GG)	24.85	0.0016	67.8%	35.2%–84%
Asian	Additive (A *vs* G)	7.55	0.1091	47%	0%–80.6%
	Recessive (AA *vs* AG+GG)	1.54	0.214	35.2%	—
	Dominant (AA +AG *vs* GG)	4.41	0.0358	77.3%	—
	Homozygous (AA *vs* GG)	1.69	0.1932	40.9%	—
Caucasian	Additive (A *vs* G)	467.45	< 0.0001	96.8%	95.8%–97.5%
	Recessive (AA *vs* AG+GG)	18.19	0.0027	72.5%	36.7%–88.1%
	Dominant (AA +AG *vs* GG)	19.68	0.0014	74.6%	42.3–88.8%
	Homozygous (AA *vs* GG)	19.83	0.0013	74.8%	42.8%–88.9%

— represent the missing data because that there were few studies in Asian population 95% CI of I^2^ did not can be estimate.

We used meta-regression to further investigate the potential sources of heterogeneity, including publication year, country, ethnicity, sample size, bias of sample size, type of control population and SNP genotyping techniques. We found that publication year, sample size and bias of sample size were significant, and these factors may be the sources of the observed heterogeneity (Supplementary Table 1). The absence of significant effects of country, ethnicity and type of control population implied that these factors cannot be cause of the heterogeneity among the studies.

### Meta-analysis

**Figure 2:**
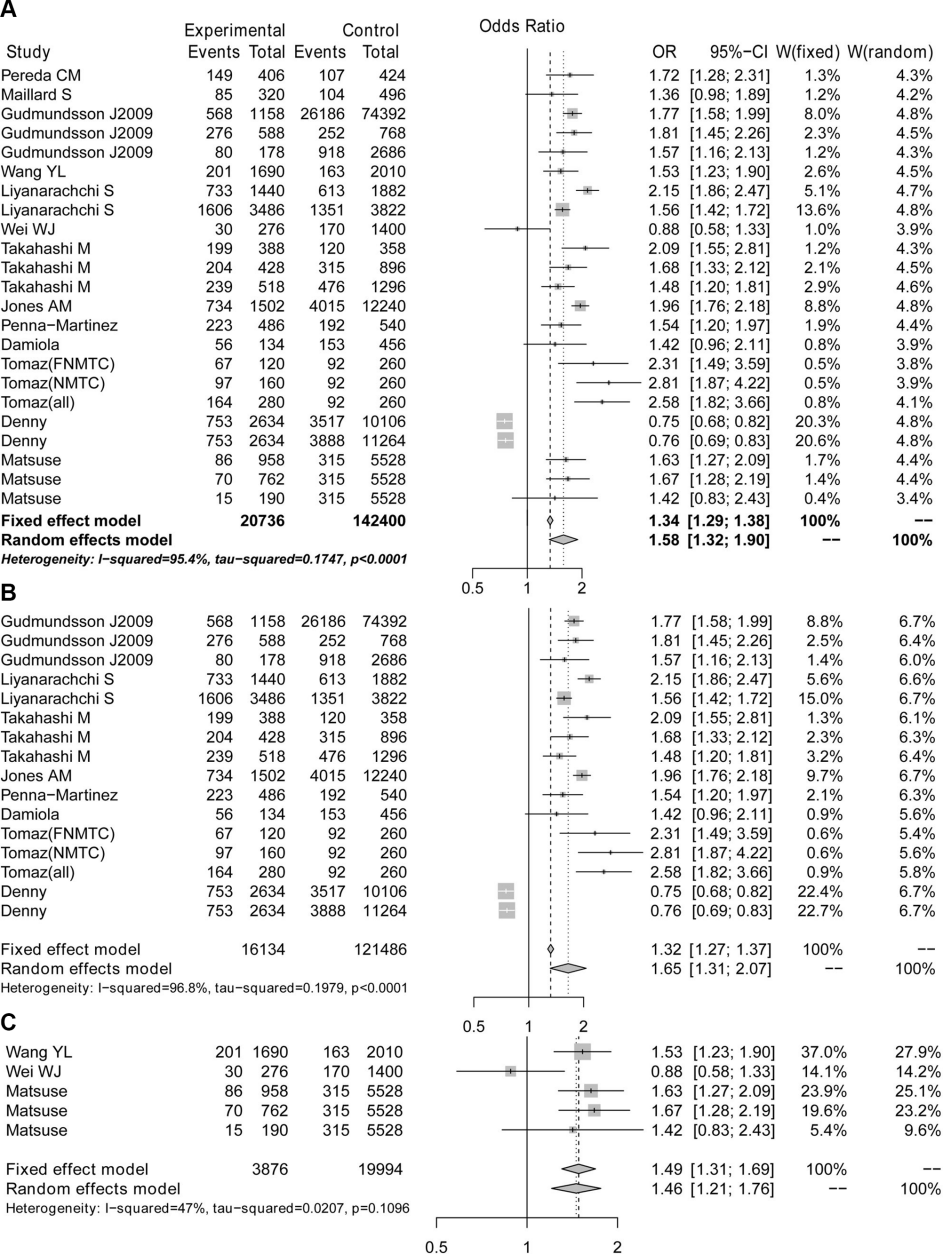
The forest plot for A *vs* G of the rs965513 polymorphism (**A**) All populations. (**B**) Caucasian populations. (**C**) Asian populations.

**Figure 3:**
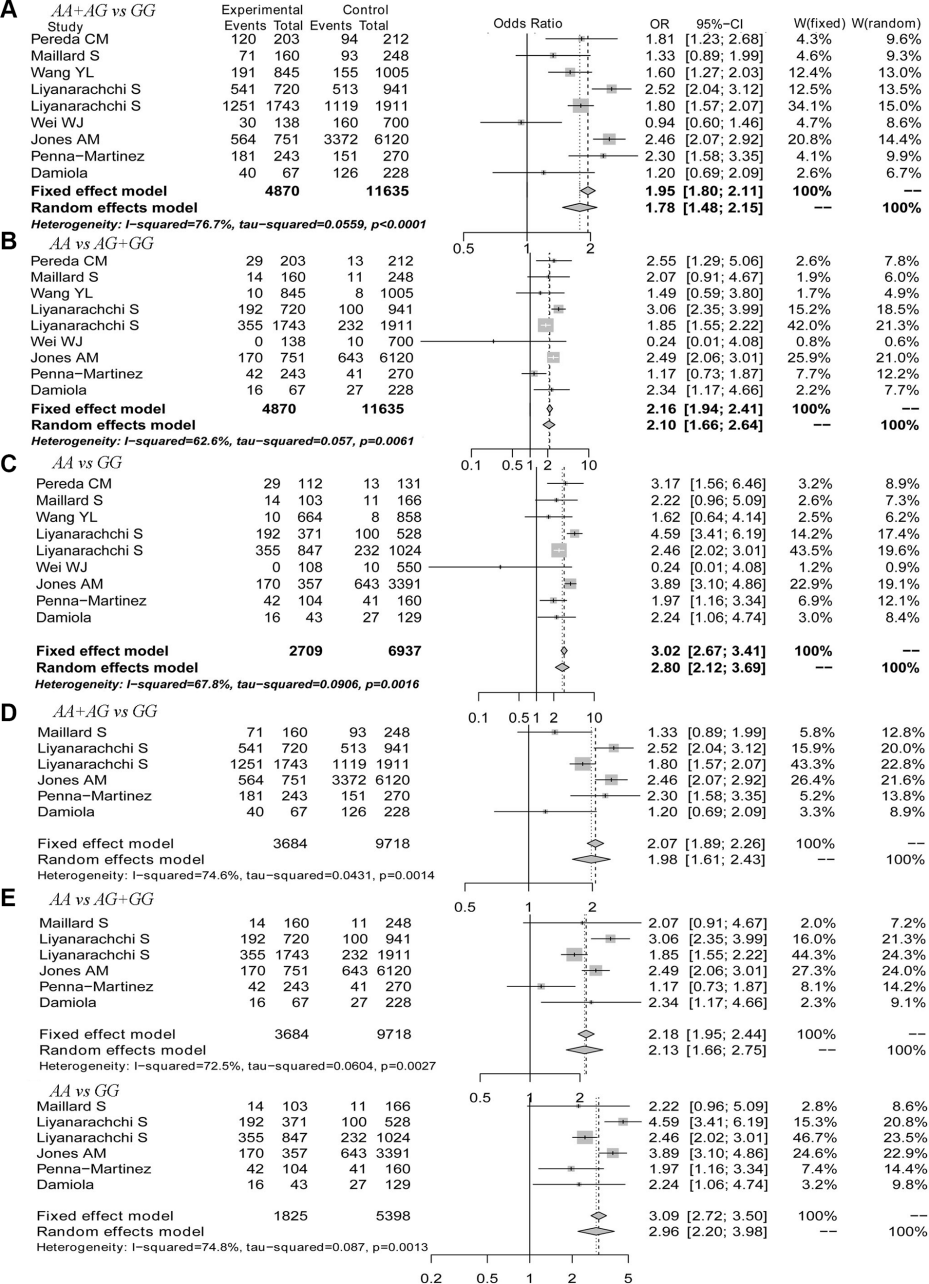
The forest plot for genotypes of the rs965513 polymorphism (**A**) Dominant model in all populations. (**B**) Recessive model in all populations. (**C**) Homozygous model in all populations. (**D**) Dominant model in Caucasian populations. (**E**) Recessive model in Caucasian populations. (**F**) Homozygous model in Caucasian populations.

We performed a meta-analysis to calculate the overall ORs using the random effect model in all populations and in Caucasian populations and using the fixed effect model in Asian populations based on heterogeneity analysis. The risk of thyroid cancer associated with the A allele was 1.58-fold that of the G allele (Figure 2A, OR = 1.58, 95% CI 1.32–1.90). Moreover, we found that Caucasian populations had higher risk than Asian populations (Figure 2B and 2C, OR = 1.65 *vs.* 1.49), and this result could be strengthened by including more studies. Because 9 of the 23 selected studies provided the number of rs965513 genotypes or provided sufficient data to calculate the number of rs965513 genotypes, meta-analyses using dominant, recessive and homozygous models were conducted among these 9 studies. The association between rs965513 and thyroid cancer was also significant in the dominant model (Figure 3A, AA + AG *vs.* GG, OR = 1.78, 95% CI 1.48 – 2.15), the recessive model (Figure 3B, AA *vs.* AG + GG, OR = 2.10, 95% CI 1.66–2.64), and the homozygous model (Figure 3C, AA *vs.* GG, OR = 2.80, 95% CI 2.12–3.69). In addition, the genotype AA was most strongly associated with risk of thyroid cancer in Caucasian populations (Figure 3D–3F). Only two studies provided the number of rs965513 genotypes, so the risks of genotypes in Asian populations could not be estimated.

### Sensitivity and publication bias analysis

**Figure 4:**
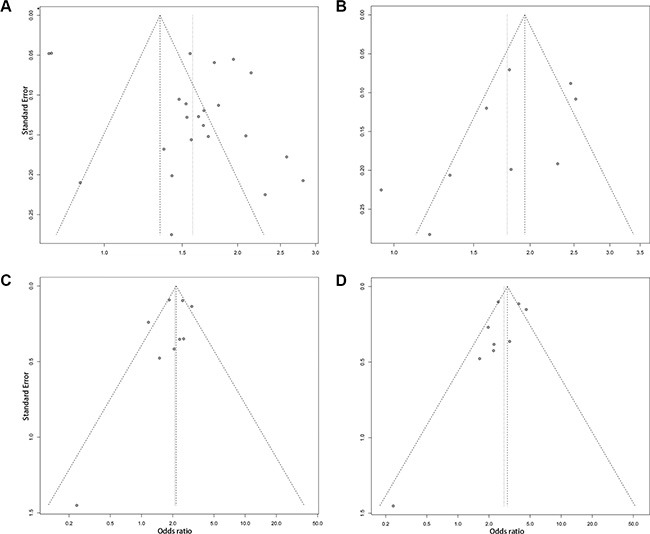
The funnel plots for publication bias analysis of the rs965513 polymorphism (**A**) Additive model. (**B**) Dominant model. (**C**) Recessive model. (**D**) Homozygous model.

We performed a one-way sensitivity analysis to evaluate the robustness of the results of this meta-analysis. The pooled ORs from different populations were not influenced by removal of one study under four genetic models (Supplementary Tables 2 and 3), suggesting that the results of this meta-analysis are stable. Begg's and Egger's tests were performed to evaluate publication bias. Although the funnel plots of the additive model were asymmetrical inverted funnels (Figure 4A), the results of both Begg's test and Egger's test were not significant (Table 3). In addition, the funnel plots of the dominant, recessive and homozygous models are symmetrical inverted funnels (Figure 4B–4D), which suggests no significant publication bias. The above results suggest that the conclusions of our meta-analysis are credible and stable.

**Table 3:** The result of Begg and Egger's tests

Risk model	Egger' s test		Begg' s test	
	T statistic	*P* value	Z statistic	*P* value
Additive	2.07	0.05086	−0.87	0.38
Recessive	−0.85	0.42	−0.28	0.36
Dominant	−1.44	0.19	−0.56	0.04
Homozygous	−1.31	0.23	−0.22	0.48

## DISCUSSION

FOXE1, which is also called TTF2 (Thyroid transcription factor 2), is a transcription factor involved in thyroid gland development (thyroid formation, migration and morphogenesis control [20–23]) and in the maintenance of differentiation in the thyroid [24] and which is highly expressed in thyroid follicular cells [25, 56]. According to Goldgar DE and Eng C, multiple low- to moderate-penetrance genes (LPGs) interacting with each other and with the environment may result in thyroid cancer [27, 28]. FOXE1 is a likely LPG in this content because FOXE1 is the center of a regulatory network of transcription factors and cofactors that initiate thyroid differentiation [22]. The influence of FOXE1 on thyroid cancer has been investigated by Rihab Kallel [24]. The llelic, genotypic and phenotypic analyses strongly suggested that the length of the alanine stretch in FOXE1 modulates genetic susceptibility to papillary thyroid cancer. They reported that the 16-Ala allele and homozygous 16/16 genotype showed increased risk of thyroid cancer development. However, subjects with the 14-Ala allele seemed to be protected against the occurrence of this pathology [24]. The presence of rs965513 near FOXE1, located on 9q22, showed a ~1.8-fold odds ratio of PTC risk through genome-wide association. He et al. investigated the molecular mechanism by which rs965513 regulated the expression of FOXE1, generating susceptibility to thyroid cancer [29]. They found rs965513 located in a linkage disequilibrium block ~33 kb including at least three regulatory elements functioning as enhancers. The region overlapped with the promoter region of FOXE1, and the variability of genotypes was associated with differential activity levels of an enhancer, further leading to variations in FOXE1 expression that resulted in altered risk of thyroid cancer.

The significant association between the rs965513 polymorphism and thyroid cancer was first identified by Gudmundsson J in 2009 [7]. Over the following few years, subsequent studies have continued to explore this association and have reported both consistent and conflicting results. Geng et al. reviewed 10 studies and observed that the A allele of rs965513 had a 1.31-fold risk of thyroid cancer [30]. However, Kang et al. evaluated the association of 12 SNPs in FOXE1 and PTC, and reported that rs965513 showed no association with PTC [31]. It is important to assess the genetic architecture of the rs965513 polymorphism across different ethnicities and populations. There were several similar meta-analyses that have assessed the risk of rs965513 in PTC [31–35]. However, we used a larger sample size of 13 articles corresponding to 23 studies (20,736 cases *vs*. 142,400 controls) found in PubMed and Google Scholar to reevaluate this association, which will help to accurately assess the risk of rs965513 in PTC. Kang J. et al. included 8 studies corresponding to 2,085 cases and 10,341 controls [31]. Zhuang Y. et al. included 13 studies involving 8491 cases, 103,218 control and 629 family members [32]. Ai L et al. included 6 studies and a total of 52,363 individuals (5,193 cases vs. 47,170 controls) [33]. Zhu et al. included 14 studies and a total of 9828 subjects [34]. Gao et al. included 16 studies and 8119 cases vs. 66,936 controls [35]. Moreover, we evaluated the risk of thyroid cancer with rs965513 under several genetic models including the additive, recessive, dominant and homozygous models, to assess the increased levels of PTC risk under different genotypes. Zhuang et al. evaluated the allelic, dominant and recessive models [32]. Ai L et al. performed meta-analysis using additive model [33]. Zhu et al. performed meta-analysis using the additive, heterozygous and homozygous models [34]. Lastly, we considered differential associations between rs965513 and PTC in different populations and performed stratified meta-analysis separately in Asian and Caucasian populations. Ai L et al. performed meta-analysis in a mixed population [33].

In our meta-analysis, the genetic heterogeneity of rs965513 among the selected studies was evaluated, and significant heterogeneity was observed in the additive, dominant, recessive and homozygous models. The genetic heterogeneity may be caused by differences in the publication year, sample size and bias of sample size through meta-regression. Because more studies corresponded to Caucasian populations, significant heterogeneity was observed in Caucasian populations but not in Asian populations. Next, meta-analyses were conducted separately for Caucasian, Asian and all populations. Our results showed that the A allele of rs965513 had a 1.58-fold risk of thyroid cancer in all populations, a 1.65-fold risk (95% CI 1.31–2.07)) in Caucasian populations and a 1.49-fold risk in Asian populations. Compared to the dominant and recessive models, the homozygous model showed the highest odds ratio (OR = 2.80, 95% CI 2.12–3.69) in all populations and in the Caucasian populations. The one-way sensitivity analysis suggested that the results of this meta-analysis were stable. We found that the Caucasian populations had higher risk than Asian populations, which further supports the previous findings.

In addition to the rs965513 polymorphism, two other variants of FOXE1, rs1867277 and rs71369530, are also significantly associated with thyroid cancer [4]. The variant rs1867277 is located within the 5′ untranslated region (UTR) and is involved in the allele-specific transcriptional regulation of FOXE1 through recruitment of the transcription factors USF1/USF2 [4]. Jones et al. reconstructed haplotypes at these two loci (rs965513 and rs1867277) and estimated the ORs associated with having each of the three possible risk haplotypes compared to the non-risk haplotype. They reported that carrying the haplotype with both risk alleles significantly increases the risk of thyroid cancer, while carrying haplotypes with a single risk allele at either rs965513 or rs1867277 somewhat increases the risk [14]. The variant rs71369530 is a poly-alanine expansion in the FOXE1 coding region. Martyn's study revealed that the poly-alanine expansion of FOXE1 (rs71369530) is significantly associated with PTC in Caucasian subjects (OR = 2.23, 95% CI 1.42–3.50) [36].

We conducted meta-analyses using additive, dominant, and recessive models at the same time for more powerful results. However, our study has certain limitations. We attempted to obtain exact genotype numbers from all studies used in our analysis for the dominant and recessive models, but only some of the genotype numbers were available [4, 8, 10–16]. Future studies can supplement our results using dominant and recessive models.

## MATERIALS AND METHODS

### Literature search

In stage 1, we searched PubMed and Google Scholar to select all possible studies with key words including ‘rs965513’ and ‘thyroid cancer’ or ‘FOXE1’ and ‘thyroid cancer’. The literature search was updated on April 7, 2015. Then, in stage 2, we used Google Scholar (http://scholar.google.com/) to query the articles citing these studies and all of the references therein as identified using PubMed. We selected only published articles written in English.

### Inclusion criteria

The studies that were selected met the following criteria: (1) the study must use a case-control design; (2) the study evaluated the association between rs965513 polymorphism and thyroid cancer; (3) the study provided the number of rs965513 genotypes or (4) provided sufficient data to calculate the number of rs965513 genotypes; and (5) the study provided an OR with 95% CI as well as the *P* value or (6) the study provided sufficient data to calculate the OR and 95% CI.

### Data extraction

We extracted the following information from every study: (1) the name of the first author; (2) the year of publication; (3) the population or ethnicity; (4) the numbers of cases and controls; (5) the genotype number of rs965513 polymorphisms in cases and controls; (6) the number of rs965513 genotypes or (7) information needed to calculate the numbers of rs965513 genotypes; (8) the OR with 95% CI or (9) information needed to calculate the OR and 95% CI. All related calculations were completed using R software.

### Genetic model

The rs965513 polymorphism includes the two alleles G and A, of which A is the minor allele. A is assumed to be the high-risk allele and G the low-risk allele. We selected the additive, dominant, recessive and homozygous genetic model for further meta-analysis. The additive model can be described as the A allele versus the G allele [37].

### Heterogeneity test

Cochran's *Q* test was used to evaluate the genetic heterogeneity among the included studies, which approximately follows a χ^2^ distribution with k-1 degrees of freedom (k stands for the number of studies for analysis). I^2^ = (Q-(k-1)) ÷ Q × 100%, ranging from 0 to 100% [38], was also used. I^2^ is a measure of heterogeneity and is a statistic that indicates the percentage of variance in a meta-analysis that is attributable to study heterogeneity [39]. Low, moderate, large and extreme heterogeneity corresponded to 0–25%, 25–50%, 50–75% and 75–100%, respectively [39]. The significance levels for heterogeneity are defined as *P* < 0.01 and I^2^ > 50%. Meta-regression was used to investigate the potential sources of heterogeneity and was performed using the metafor package in R software.

### Meta-analysis

For case in which there is no significant heterogeneity among the included studies, the pooled OR was calculated using the fixed effect model; otherwise, the OR was calculated using random-effect model. The Z test was used to determine the significance of ORs. All statistical tests for heterogeneity and meta-analysis were performed using the meta package in R software.

### Publication bias analyses

Funnel plots were used to evaluate the potential publication bias [40]. Begg's and Egger's tests were used to evaluate the asymmetry of the funnel plot [40]. It is assumed that in the absence of publication bias, the largest study will be plotted near the average, and smaller studies will be spread evenly on both sides of the average, creating a roughly funne-shaped distribution. Deviation from this shape can indicate publication bias. The publication bias analyses were performed using the metafor package in R software.

## SUPPLEMENTARY FIGURES AND TABLES


