# Supplementary material for: rs965513 polymorphism as a common risk marker is associated with papillary thyroid cancer
Source: Oncotarget. 2016 May 12;7(27):41336–45. doi: 10.18632/oncotarget.9324 (PMC5173063; doi:10.18632/oncotarget.9324)
Supplement: Supplementary file 1 [file oncotarget-07-41336-s001.pdf]

rs965513 polymorphism as a common risk marker is associated with papillary thyroid cancer

Supplementary Materials

Supplementary Table S1: Results of meta-regression

	Year	Country	Ethnicity	Sample size	Bias of sample size	Control population	Genotyping
Estimate	0.245	0.001	–0.809	–0.508	1.830	–0.462	0.255
P value	< 0.001	0.986	0.108	< 0.001	< 0.001	0.348	0.216

Supplementary Table S2: Sensitivity analysis of additive model

All population				
**Study excluded**	**OR**	**95% CI**	**I^2**	***p***
None	1.58	1.32–1.90	95.4%	< 0.0001
Pereda CM	1.58	1.31–1.90	95.6%	< 0.0001
Maillard S	1.59	1.32–1.92	95.6%	< 0.0001
Gudmundsson J2009	1.56	1.28–1.92	95.8%	< 0.0001
Wang YL	1.59	1.31–1.91	95.6%	< 0.0001
Liyanarachchi S	1.56	1.28–1.91	95.2%	< 0.0001
Wei WJ	1.62	1.35–1.95	95.6%	< 0.0001
Takahashi M	1.56	1.28–1.91	95.9%	< 0.0001
Jones AM	1.57	1.30–1.89	95.1%	< 0.0001
Penna-Martinez	1.58	1.31–1.91	95.6%	< 0.0001
Damiola	1.59	1.32–1.91	95.6%	< 0.0001
Tomaz	1.48	1.23–1.79	95.8%	< 0.0001
Denny	1.72	1.59–1.87	61.1%	0.0001
Matsuse	1.58	1.30–1.93	96%	< 0.0001
**Asian**				
None	1.49	1.31–1.69	47%	0.1096
Wang YL	1.46	1.24–1.71	60.1%	0.06
Wei WJ	1.58	1.38–1.81	0	0.92
Matsuse	1.35	1.12–1.63	81.4%	0.02
**Caucasian**				
None	1.65	1.31–2.07	96.8%	< 0.0001
Gudmundsson J2009	1.63	1.25–2.13	97.2%	< 0.0001
Liyanarachchi S	1.62	1.25–2.11	96.8%	< 0.0001
Takahashi M	1.63	1.26–2.12	97.4%	< 0.0001
Jones AM	1.63	1.28–2.06	96.6%	< 0.0001
Penna-Martinez	1.65	1.30–2.10	97%	< 0.0001
Tomaz	1.50	1.18–1.92	97.2%	< 0.0001
Damiola	1.66	1.31–2.10	97%	< 0.0001
Denny	1.82	1.66–1.99	63.3%	0.0007

Supplementary Table S3: Sensitivity analysis of recessive model

All population						
	Recessive		Dominant		Homozygous	
**Study excluded**	**OR**	**95% CI**	**OR**	**95% CI**	**OR**	**95% CI**
None	2.10	1.66–2.64	1.7797	1.4756–2.1465	2.7995	2.1244–3.6889
Pereda CM	2.06	1.60–2.64	1.7700	1.4428–2.1714	2.7451	2.0332–3.7063
Maillard S	2.09	1.63–2.68	1.8357	1.5118;–2.2290	2.8417	2.1206–3.8081
Wang YL	2.13	1.68–2.71	1.8010	1.4618–2.2189	2.9052	2.1891–3.8555
Liyanarachchi S	2.15	1.83–2.52	1.6378	1.2572–2.1337	2.4953	1.7174–3.6255
Wei WJ	2.13	1.70–2.67	1.9071	1.6096–2.2596	2.8804	2.2095–3.7550
Jones AM	1.98	1.48–2.66	1.6894	1.3869–2.0578	2.5723	1.8674–3.5432
Penna-Martinez	2.2929	1.8619–2.8235	1.7254	1.4085–2.1136	2.9439	2.1987–3.9417
Damiola	2.0704	1.6121–2.6590	1.8332	1.5145- 2.2189	2.8466	2.1200–3.8221
**Asian**						
None	1.0858	0.4745–2.4842	1.4200	1.1567; 1.7432	1.1608	0.5088–2.6485
**Caucasian**						
None	2.1073	1.5959–2.7826	2.0795	1.6975–2.5476	3.0431	2.2083–4.1935
Liyanarachchi S	2.1835	1.8347–2.5987	2.2602	1.9542–2.6142	3.3438	2.7325–4.0919
Jones AM	1.9638	1.3240–2.9128	1.9613	1.7631- 2.1817	2.7784	1.8319–4.2137
Penna-Martinez	2.2667	2.0168–2.5476	2.0373	1.6110–2.5763	3.3126	2.3498–4.6698
